# Droplet-interface-bilayer assays in microfluidic passive networks

**DOI:** 10.1038/srep09951

**Published:** 2015-04-24

**Authors:** Bárbara Schlicht, Michele Zagnoni

**Affiliations:** 1 Centre for Microsystems and Photonics, Electronic and Electrical Engineering, University of Strathclyde, Glasgow, G1 1XW, UK

## Abstract

Basic biophysical studies and pharmacological processes can be investigated by mimicking the intracellular and extracellular environments across an artificial cell membrane construct. The ability to reproduce *in vitro* simplified scenarios found in live cell membranes in an automated manner has great potential for a variety of synthetic biology and compound screening applications. Here, we present a fully integrated microfluidic system for the production of artificial lipid bilayers based on the miniaturisation of droplet-interface-bilayer (DIB) techniques. The platform uses a microfluidic design that enables the controlled positioning and storage of phospholipid-stabilized water-in-oil droplets, leading successfully to the scalable and automated formation of arrays of DIBs to mimic cell membrane processes. To ensure robustness of operation, we have investigated how lipid concentration, immiscible phase flow velocities and the device geometrical parameters affect the system performance. Finally, we produced proof-of-concept data showing that diffusive transport of molecules and ions across on-chip DIBs can be studied and quantified using fluorescence-based assays.

Over the past two decades, the steady development of microfluidic technologies has provided sophisticated methodologies in many areas of science, including the ability to integrate and multiplex bioassays. The creation of biocompatible environments and the laminar flow properties of microfluidic channel networks offer very exciting prospects for the future development of automated and high-throughput synthetic biology-based platforms.

Of particular interest for both drug discovery and biophysical research is the ability to interrogate and characterise the functional behaviour of membrane proteins in a reliable, scalable and miniaturised format. To this end, either live cell systems or a simplified synthetic environment that mimics that of a natural cell membrane can be used. Microfluidic solutions are already commercially available (e.g. from Fluxion, Nanion Technologies, Sophion and Ionera) that use electrophysiology techniques to measure the activity across biological membranes, using either whole live cells^1^ or by inserting ion channels in artificial lipid bilayers^2^. The latter approach is particularly interesting as it allows the effect of cellular parameters (e.g. membrane composition, pH and ion channel type) to be separately investigated, this having strong implications for carrying out novel types of experiments, for advancing biological knowledge related to membrane proteins and for facilitating the design and testing of more effective drugs.

Microfluidic solutions have been proposed to create artificial cell membranes using immiscible fluids in silicon and polymer substrates, where suspended lipid bilayers were formed solely by fluid phase manipulation^3^,^4^,^5^,^6^,^7^. Alternatively, phospholipid stabilised water-in-oil (psW/O) droplets have been used to create artificial cell membranes (known as droplet-interface-bilayers^8^ - DIBs). DIB formation is achieved when two psW/O droplets come into contact and the lipid molecules at each droplet interface interact spontaneously self-assembling into a lipid bilayer. Initially, DIB based assays have been developed by manually bringing droplets into contact but, in recent years, more efforts have been devoted to establishing similar assays using miniaturised systems that relied on micromanipulators, microgeometries and dielectrophoresis^9^,^10^,^11^,^12^,^13^,^14^,^15^,^16^ or by interfacing psW/O droplets with agarose gel layers^17^,^18^. However, these approaches present limitations due to either a lower throughput than automated patch-clamp systems or necessitate procedures that require manual intervention. Additionally, microfluidic systems based on arrays of individual giant-unilamellar-vesicles (GUVs) have also been reported^19^. Although GUVs are a useful model for membrane morphology studies, their formation takes place outside the device and the inner vesicle compartment cannot be easily accessed or modified.

To overcome these challenges we have developed a microfluidic solution that combines into a single device the throughput typical of droplet microfluidic systems^20^ with the high degree of control over droplet positioning obtained from bespoke microchannel networks^21^. The system facilitates by design the precise and automated positioning of droplets within microchannels, offering new solutions for developing DIB assays in an automated fashion using fluorescence microscopy. DIBs are formed upon contact between two psW/O microdroplets encapsulating an appropriate mix of proteins/compounds where one droplet mimics the intracellular environment and the other represents the extracellular environment. Furthermore, the implementation of fluorescence-based assays in DIB format is directly compatible with the throughput of droplet technologies and reduces the complexity and costs associated with electrophysiology procedures.

In this work, a droplet-microfluidic system is presented for maximising the yield of formation of arrays of DIBs in a scalable format, integrating the formation and desired positioning of psW/O droplets. By encapsulating the desired cocktail of liposomes, buffers, molecules and peptides into the psW/O droplets, a large number of DIBs were characterized using fluorescence-based assays. Our investigation has identified the key parameters for enabling the reliable formation and storage of DIB arrays, as well as the relationship between the throughput of readout and the process dynamics occurring across a DIB.

## Methods

Materials. All chemicals were purchased from Sigma-Aldrich (UK) unless otherwise stated. 1,2-Diphytanoyl-sn-glycero-3-phosphocholine (DPhPC) and 1,2-dioleoyl-sn-glycero-3-phosphocholine (DOPC) were purchased from Avanti Polar Lipids (Alabama, US). Fluo-8 K^+^ Salt was purchased from Abcam Biochemicals (Cambridge, UK).

Device fabrication. Microfluidic devices were fabricated in polydimethylsiloxane (PDMS) (Sylgard 184, Dow Corning, US) using standard soft lithography techniques. Silicon masters were produced using SU8 photoresist (3000 series, MicroChem, US) on a silicon wafer following the manufacturer’s protocol. A layer of SU8 3035 was spun to produce a final resist thickness of 50 µm. The resist was exposed through a photomask (JD Photo-Tools, UK) to UV light and was developed in MicroPosit EC solvent (Rohm and Haas, US). To prevent PDMS adhesion to the resulting silicon master, the silicon surface was silanized by vapour deposition of 1H,1H,2H,2H-perfluorooctyl-trichlorosilane for 1 hour. PDMS was then poured onto the silicon master at a 10:1 ratio of base to curing agent, degassed in a vacuum desiccator chamber and cured at 70°C for at least 3 hours. The PDMS devices were then peeled from the mould, cut to the desired size and holes were punched to obtain the inlet and outlet ports. PDMS devices were then cleaned and irreversibly bonded to glass microscope slides using oxygen plasma. Bonded devices were then flushed with Sigmacote and air to render the channels hydrophobic.

Lipid and liposome preparation. Asolectin (a lipid mixture from soybean), DOPC or DPhPC were used for DIB formation. An aliquot of lipids in chloroform was evaporated under a stream of nitrogen and subsequently dried for 2 hours in a desiccator. The lipid film was then rehydrated to the desired concentration in either hexadecane or the desired buffer. Lipid concentrations ranging from 2 to 10 mg/mL were tested, all of which are above the critical micelle concentration (CMC). When dissolved in buffer, the samples were extruded 21 times through a 100 nm polycarbonate membrane (Avanti Polar Lipids) to produce unilamellar lipid vesicles. For DIB leakage experiments, alternating droplets containing non‐fluorescent (10 mM HEPES, 200 mM KCl, pH 7.4 - donor droplet) or fluorescent buffers (100 µM fluorescein or 100 µM calcein, 10 mM HEPES, 200 mM KCl, pH 7.4 - acceptor droplet) were used. For DIB ion channel experiments, 5 mg/mL DPhPC in hexadecane and alternating droplets containing 10 mM HEPES, 20 μM EDTA, 1 M CaCl_2_, 2 µg/ml α-Haemolysin at pH 7.4 (donor droplet) and 10 mM HEPES, 333 μM EDTA, 2 M KCl, 250 µM Fluo-8 at pH 7.4 (acceptor droplet) were used.

Coalescence experiments and fluorescence-based assays. Phospholipid stabilised water-in-oil (psW/O) droplets were arrayed and stored within “shift-register” structures^22^ forming arrays of DIBs. The structure of a droplet shift register is segmented in three parts: one main channel and two side gutters. The gutters and the main channel are separated by pillars that allow the flow of the continuous phase through, facilitating droplet arraying and trapping within the main channel (Figure S1 in ESI).

For droplet coalescence experiments, asolectin at 5 lipid concentrations was used −2, 4, 5, 8 and 10 mg/ml. For each concentration, lipids were tested when dispersed only in the oil phase or only in the buffer phase as liposomes or in equal concentration in both phases. For these experiments, psW/O droplets were obtained using a device with a single T-junction and rectangular pillars within the registers, as either droplet alternation or long-term storage was not required. Droplet arraying and coalescence behaviour were monitored and recorded within the registers, varying droplet velocities in the range 10–3,000 µm/s. Any occurrence of coalescence within any register (due to resting or moving droplets) was counted as a coalescing event.

**Figure 1:**
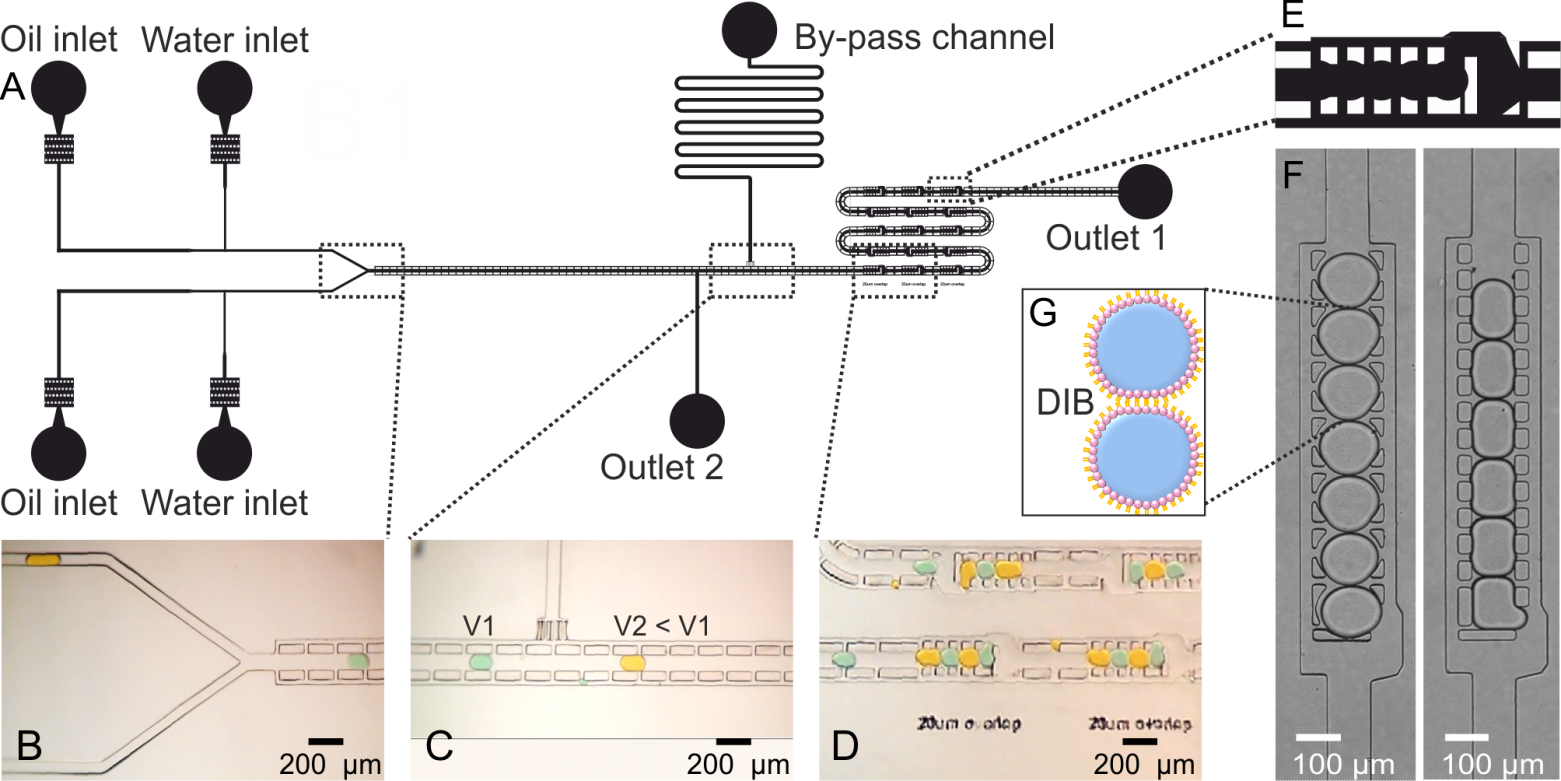
Device geometry. (A) Schematic drawing of the microfluidic device developed. A double T-Junction was used to produce droplets encapsulating different buffers. Images from supplementary movies: (B) a Y-Junction enabled droplet alternation; (C) the channel structure and a by-pass channel were designed to divert the oil phase and reduce droplet velocity after formation and before the register area; (D) a series of droplet traps (shift registers) had an optimised pattern to maintain droplets stored in the absence of flow. (E) Schematic drawing of a shift register that allows droplets to lock within the pillars in the absence of oil flow for long-term droplet storage. (F) Experimental images showing the difference between droplet arrangement in long shift registers with different shapes of pillars. The pillar structure on the left allowed droplets to remain trapped within the register when the oil flow was stopped, while droplets on the right hand image tended to backflow or move outside the registers upon detaching the tubing at the inlets. (G) Schematic representation of a DIB. As the tails of two lipid monolayers come into contact, a DIB is formed.

For leakage and ion channel experiments, psW/O droplets encapsulating fluorescent and non-fluorescent buffers were obtained using a device with a double T-junction and a pillar structure that allowed droplets to be “locked” within the register in the absence of flow to aid droplet storage over time, obtaining arrays of droplets in ABAB configuration (Figure 1). Once all the registers were completely filled with droplets, the fluid flow was stopped and tubing disconnected. Droplets were then monitored every 1–5 minutes for the duration of the experiment (40 min to 2 hours).

Microfluidic device operation. A commercially available pressure control system (MFCS 4C, Fluigent) was used to independently drive the phases into the device, applying pressure patterns typically in the range 0–150 mbar. The system allowed the prompt generation of on-demand droplets at each T-junction by adjusting the pressure of each phase simultaneously via computer controlled software.

An inverted microscope (Axiovert A1, Zeiss) was used for all experiments and images acquired using either a CMOS Genie HM1024 camera (Teledyne Dalsa) via in house written Labview software or an EMCCD LucaR camera (Andor Technologies) via Andor Solis software. Objective lenses of 2.5x, 5x, 10x or 20x were used. Fluorescence images were acquired using a fluorescein isothiocyanate filter (FITC) with 200 ms exposure time. Fluorescence count from the droplets was obtained by averaging the intensity values of a region of interest of 50×50 pixels in the centre of the droplet. ImageJ software (v1.46r) was used to analyse and process recorded images. To test the effect of droplet coalescence at different lipid concentrations, videos (up to 200 frames/s) were taken of the device in operation (Movies S1A-C in ESI) with droplets travelling at different velocities (Movies S2, S3, and S4 in ESI). The videos were subsequently examined using ImageJ and droplet velocity measured for a range of applied pressures and lipid concentrations.

## Results

We present a droplet microfluidic platform that enables the miniaturisation and automation of DIB techniques allowing both short- and long-term fluorescent assays to be performed. Key characteristics of the proposed system are the use of nL-sized psW/O droplets which minimise assay costs and a bespoke microfluidic network which ensures DIB formation in a computer controlled format, allowing fast and reconfigurable start/stop of experimentation.

### Device Geometry

We designed and validated a microfluidic structure capable of the following integrated serial functions: droplet production, droplet alternation, in-line change of droplet velocity, DIB formation and storage of DIB arrays (Figure 1).

The microfluidic design comprises a double T-junction (for droplet formation), a Y-junction (for droplet alternation, Figure 1B), a bypass channel (to adjust the velocity of the droplets, Figure 1C) and a series of microfluidic shift registers^22^ (for droplet trapping, DIB formation and storage, Figures 1D, E & F). Briefly, a droplet shift register is a microfluidic structure that allows droplets to flow within a central channel formed by pillars while the oil phase also flows outside the pillars. This allows a balance between hydrodynamic and interfacial tension forces to be established across the length of the register (Figures S1 in ESI). Such force balance determines how many droplets can be stored within a register. When the register is filled with droplets, an additional droplet forces the first one in line at the downstream side to exit the register through an aperture, creating a serial shift of the drops stored within the pillars (Movie S1C in ESI). Hence, DIB formation is achieved when any two psW/O droplets come into contact within a register and the lipid molecules at each droplet interface self-assemble into a lipid bilayer (Figure 1G). The register structures can be tuned to form the desired droplet train length (i.e. DIB array) and enable from one to an array of DIBs to be formed in series (Figure S2 in ESI).

Droplet trapping and storage was facilitated by the shape of the pillars within a register (Figures 1E & F), locking each droplet into position due to surface tension forces once the oil flow was stopped. Outlet 2 (Figure 1A) allowed the extraction of residual air when initially filling the device. This outlet was subsequently blocked and not used during normal device operation, thus forcing fluids through the passive network of registers. Of particular benefit for reliable DIB formation was the addition of a by-pass channel (Figure 1C). This feature facilitates control over droplet velocity, allowing a reduction in droplet speed by diverting only the continuous phase towards the outlet. Reduction in velocity ultimately increased trapping efficiency and prevented droplet coalescence (i.e. see paragraphs on DIB stability).

Examples of device operation are shown in Movies S1A-C. It is worth noting that in all experiments droplet shrinkage occurred over time, due to fluid absorption through the PDMS walls (both hexadecane and aqueous phase). To minimise this effect all devices were immersed in water for at least one hour prior to experimentation, obtaining a 16% droplet area decrease after 40 minutes (Figure S3 in ESI).

### DIB Stability

An essential requirement for robust DIB formation is that two phospholipid-enveloped droplets must come into contact without merging. For this to happen, the concentration of phospholipids adsorbed at the oil-water interface must be sufficiently high to prevent coalescence of two aqueous droplets upon contact. Both thermodynamic and fluid-dynamic effects will influence the lipid behaviour at a droplet interface within a microfluidic flow.

**Figure 2:**
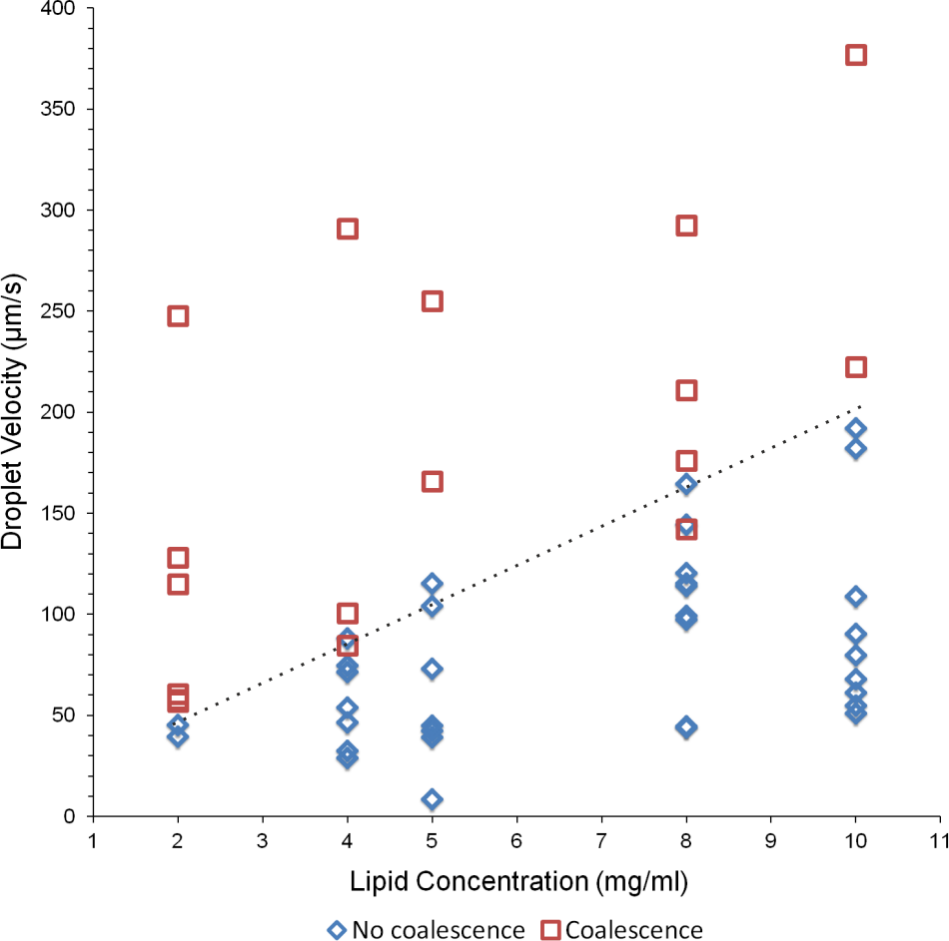
Relationship between droplet velocity and lipid concentration. The aqueous phase was a physiological buffer, the oil phase was hexadecane and asolectin was dissolved in one phase only or in both. At higher lipid concentrations and lower droplet velocities, droplets became less susceptible to coalescence upon contact within the registers. The dashed line shows an approximately linear dependence between lipid concentrations and droplet velocity.

To investigate this condition, we characterised droplet coalescence within shift register structures as a function of lipid concentration and droplet velocity (where droplet velocity is defined here as the constant velocity of a droplet flowing in a rectangular microchannel before entering a shift register structure). For each lipid concentration tested (all above their respective CMC), the droplet diameter was kept constant by adjusting the ratio of aqueous to oil phase pressure at the inlets, thus enabling a range of droplet velocities to be obtained for similarly sized droplets. The frequency of droplet coalescence for a particular lipid concentration decreased with decreasing droplet velocity (Movie S2, S3 & S4 in ESI). Overall, a threshold velocity was observed separating coalescing droplets and non-coalescing droplets against increasing lipid concentration, a trend which can be considered linear in first approximation (Figure 2). Collisions above the threshold line exhibited a higher likelihood of droplet coalescence, whereas below this line emulsions remained stable and coalescence never occurred. This trend proved valid for different types of phospholipids (asolectin, DPhPC and DOPC), although threshold values varied (data not shown). Additionally, to investigate whether coalescence was dependent on the phase in which lipids were added, for each concentration, tests were carried out where lipids were suspended in the aqueous phase as liposomes, dissolved within the oil phase or added to both phases at the same time. No coalescence trend was identified for these different cases, indicating that coalescence was not dependent on the phase in which the lipids were present.

It is also worth noting that increasing lipid concentration resulted in a decrease of interfacial tension (Figure S4 in ESI). At lower interfacial tensions, droplets were more deformable thus lowering the trapping efficiency and effecting DIB formation.

### Fluorescent assays

**Figure 3:**
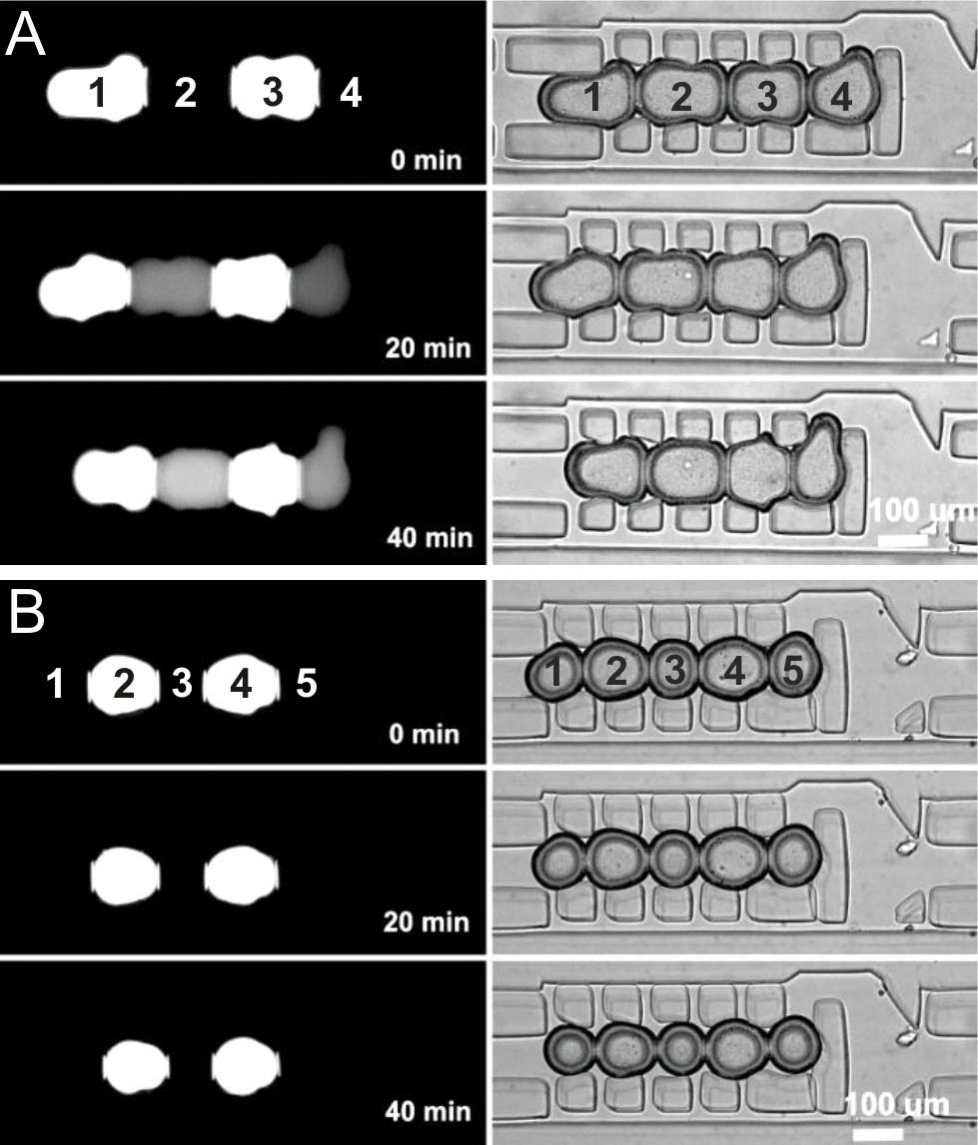
Passive molecular transport across microfluidic DIBs (A & B). Fluorescent (donor) and non-fluorescent (acceptor) droplets were trapped within registers and images where acquired every 5 minutes for 1 hour. The left hand column shows fluorescence microscopy images taken using a FITC filter. The corresponding bright field can be seen in the right hand column. 10 mg/ml asolectin was used and fluorescent droplets contained either 100 µM fluorescein (A) or 100 µM calcein (B). Fluorescein was clearly observed to leak through the lipid layer, increasing the fluorescent contents of adjacent droplets whilst calcein did not permeate through to the neighbouring droplet over the same time period.

**Figure 4:**
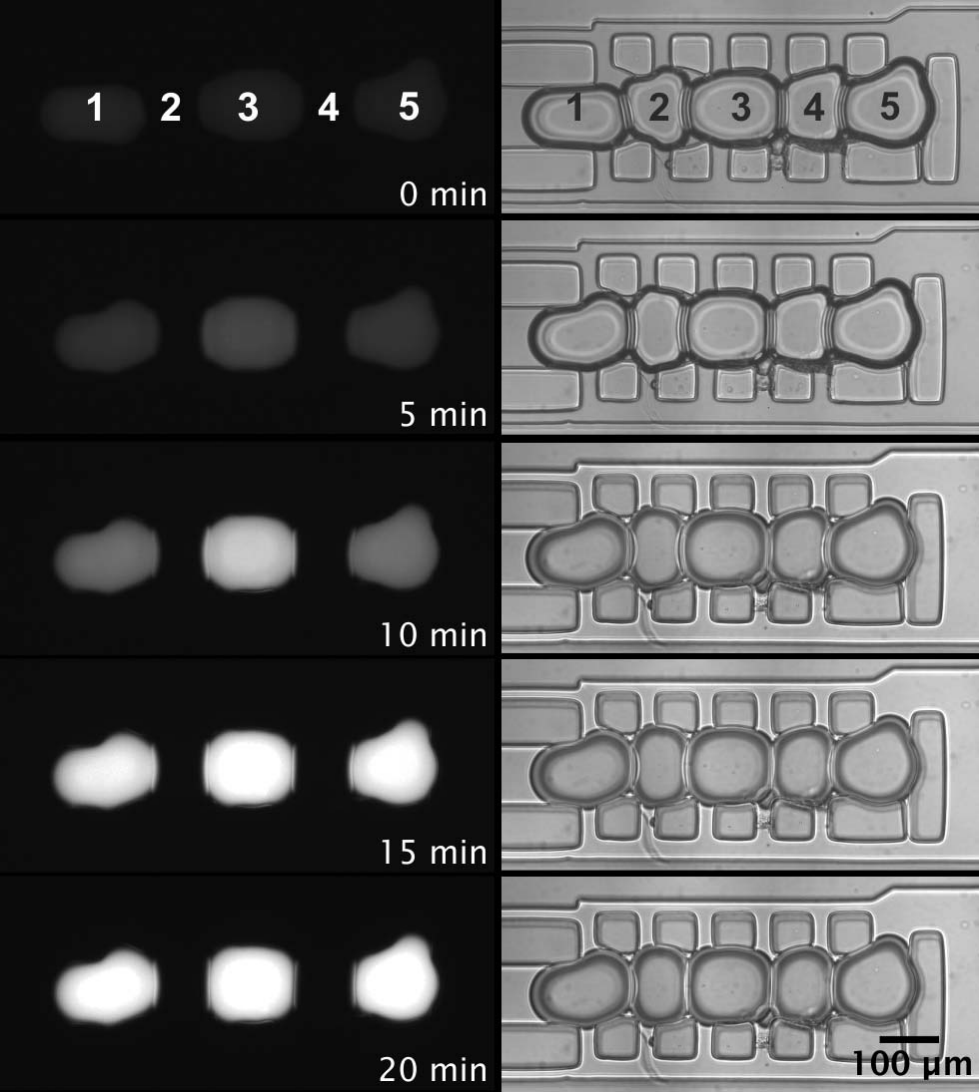
α-haemolysin pore formation in microfluidic DIBs. Acceptor droplets (1, 3 and 5) containing 250 μM Fluo-8, 10 mM HEPES, 333 μM EDTA, 2M KCl and donor droplets (2 and 4) containing, 2 μg/ml α-Haemolysin, 10 mM HEPES, 20 μM EDTA, 1M CaCl_2_ pH 7.4 were trapped in ABABA configuration. Incorporation of the α-haemolysin pore into the DIB produced a diffusive calcium flux into the neighbouring droplets. The left hand column shows fluorescence microscopy images taken using a FITC filter. The corresponding bright field can be seen in the right hand column. 5 mg/ml DPhPC was used. Movie S5 in ESI shows the increase in fluorescence intensity over time.

After DIB formation, transport through a lipid bilayer of either fluorescent dye molecules due to passive diffusion or Ca^2+^ ions across ion channels was tested, demonstrating proof-of-concept results and the suitability of the proposed microfluidic system for automated and scalable DIB-based permeation assays based on fluorescence. Furthermore, both dye leakage and ion channel experiments (Figures 3 & 4) demonstrated that DIB formation occurred, if not instantaneously, within a few minutes from droplet contact for all lipid concentrations tested. Each experiment was repeated a minimum of 3 times.

First, a membrane permeability assay^23^ was employed to characterise the leakage of fluorescent molecules through a microfluidic DIB. This assay was performed by comparing the passive transfer of fluorescein and its derivative, calcein (bis[N,N0-di(carboxymethyl)-aminoethyl] fluorescein). Molecules with a lower charge are more permeable through a lipid layer than those with a higher charge^24^,^25^. At neutral pH, the fluorescein and calcein moiety is mainly found as monoanios and dianions, with fluorescein yielding mainly monoanions and calcein yielding mainly dianions. Since monoanions are the only form that permeates the lipid bilayer, droplets containing fluorescein are expected to permeate a lipid bilayer faster than those containing calcein due to the lower charges of these species in solution. To confirm this, we formed psW/O droplets in an ABAB configuration, with one droplet encapsulating a fluorescent buffer (with either fluorescein or calcein) and its neighbour containing a non-fluorescent buffer (Figure 3). The permeation of fluorescent molecules was monitored immediately after droplet arraying and DIB formation for a period of one hour. As expected^25^, after approximately 20 minutes, fluorescein was detected leaking through the lipid interface, as the fluorescence of the acceptor droplet increased. Contrarily, no significant fluorescence increase was detected when using calcein.

Finally, the ability of a microfluidic DIB to harbour ion channels was tested. The pore forming protein α-haemolysin, a lipid bilayer spanning toxin which forms a heptameric beta-barrel structure^26^, was utilised to fluorescently detect diffusive Ca^2+^ flux (Figure 4) using Fluo-8, a bilayer-impermeable dye that increases in fluorescence when bound to calcium. Arrays of droplets in ABAB configuration were produced, with one droplet encapsulating α-haemolysin monomers in a Ca^2+^ buffer and its neighbour containing a Fluo-8 in an isosmotic K^+^ buffer. The permeation of fluorescent molecules was monitored immediately after droplet arraying and DIB formation for a period of one hour. As α-haemolysin monomers spontaneously inserted into the bilayer, an ion channel pore was formed allowing the permeation of ions, but not Fluo-8 molecules. Consequently, a stark increase in fluorescence could be observed over time as a result of the facilitated diffusion of Ca^2+^ through the α-haemolysin pores and subsequent binding to Fluo-8 (Movie S5 in ESI). From Figure 4, it can be observed that the middle droplet became fluorescent at a faster rate than the side droplets, due to the fact that two DIBs were interfaced to the Fluo-8 containing droplet. After 15 min all three acceptor droplets were seen to reach a similar level of fluorescence as Fluo-8 molecules were saturated. As a control, the same experiment was carried out with droplets lacking in α-haemolysin monomers. The process was monitored over the same time period and no detectable increase in fluorescence was observed (Figure S5 in ESI). Ion flux through DIBs varied amongst experiments due to the stochastic nature of monomer insertion and pore formation in a lipid bilayer.

## Discussion

The analysis that follows presents our findings concerning the performance of the system, the limitations of the current microfluidic design and a discussion for future improvements.

### Device geometry

Two important features of droplet microfluidics are the throughput of experiments and the potential for automation that can be achieved with the technology^20^. The exploitation of these properties is therefore very attractive for scaling up biomembrane studies and developing synthetic biology-based procedures related to artificial lipid bilayers and ion channels.

Let’s consider scalability and throughput for screening purposes. Regarding the permeation of a compound through a DIB, increasing the throughput of result by scaling up the register network can be particularly useful for either extracting statistical information from a single device using one compound or when testing a variety of compounds at the same time. However, considering the trade-off identified for lipid concentration and droplet velocity to avoid droplet coalescence (Figure 2) and the serial link between registers, it follows that the throughput of DIB experiments that can be carried out in parallel is proportional to the time that takes to fill the register network. Therefore, the throughput of significant experiments that can be carried out in parallel with the proposed system is directly related to the timescale of the process (*T_P_*) occurring across the lipid bilayer. This is important because if the permeation of a compound through a lipid bilayer (or ion channels) occurs in a faster timescale than that required for a droplet to shift along a register, a droplet content would permeate across a DIB during the filling procedure of the register network.

To illustrate this, let’s consider a system of N shift registers where only two droplets can be stored in each register (Figure S6A in ESI). In this case, a total time of T_TOT_ = (2*N*T_D_)+(N−1)*T_T_ is required to fill the *N* registers, where T_D_ is the interval of time at which a droplet reaches any register in a steady state condition and *T_T_* is the droplet travelling time between equidistant registers (condition that can be generalised to T_TOT_ = (M*N*T_D_+(N−1)*T_T_) for a register that stores *M* droplets at the same time). Importantly, by the time all *N* registers are full, due to the serial connection between the registers, a droplet (e.g. *D1* in Figure S6A in ESI) would have been in touch with any adjacent droplet for a time T_C_ = N*T_D_ during the filling procedure (resulting in T_C_ = N*(M−1)*T_D_ for a register that stores *M* droplets at the same time). Therefore, at the time of monitoring *N* experiments in parallel (i.e. all the register are full and no more droplets are produced), the permeation process taking place across the final DIB had already been occurring in previous registers during the filling procedure. This identifies a trade-off between *T_TOT_* and *T_C_* and the time constant, *T_P_*, associated with the molecular transport dynamics across a DIB.

As an example, the device used in Movies S1A-C consisted of 15 registers each containing 5 droplets (4 DIBs per register) and therefore, considering a lipid concentration of 5 mg/ml, a time of 5–20 minutes is required to fill all the registers depending on the type of lipids used (e.g. DPhPC enabled much higher velocities than asolectin, as also previously reported^15^). This effect is shown when using calcein (*T_P_≫T_TOT_*, Figure S6B in ESI) and fluorescein (*T_P_≪T_TOT_*, Figure S6C in ESI), molecules that passively cross a DIB at different rates. In the final state after filling the first nine registers, while all DIB arrays can be simultaneously monitored for calcein-filled droplets (Figure S6B in ESI), only the first two registers can be simultaneously monitored for fluorescein-filled droplets (as fluorescein had considerably leaked through DIBs during the filling procedure, Figure S6C in ESI).

In conclusion, to address scalability of operation and throughput of results, a dual approach should be undertaken where only 1 or a small number of registers should be used in the network via automatic start/stop of droplet production in the case of *T_P_<T_TOT_*, whereas due to fast dynamics of mass transport many experiments must be repeated periodically. Although the case of *T_P_ < T_TOT_* does not occur frequently, some drugs have been reported in this range^27^. Contrarily, a proportionally larger number of registers can be monitored simultaneously for conditions where *T_P_>T_TOT_*, whereas due to slow dynamics of mass transport many experiments can be monitored in parallel.

Finally, attention should also be placed on the type of carrier fluid and device material used. In our work, except for minor advantages in reducing PDMS swelling with respect to other solvents, hexadecane was primarily used as it has been consistently reported that increasing the n-alkane chain length of the oil increases the chances of obtaining oil-free conditions within a lipid bilayer^28^, thus forming DIBs with a more similar environment to that found in live cell membranes. Further advantages can also be gained by fabricating the microfluidic devices with transparent materials that do not absorb solvents and are not gas and water permeable, such as poly-methyl-methacrylate, polystyrene or glass, resolving the issues associated with temporal droplet volume variation when performing long-term assays.

### DIB stability

The observed decrease in droplet coalescence for increasing lipid concentrations is consistent with previous reports^18^ for DPhPC bilayers formed between a resting aqueous droplet in hexadecane and an agarose substrate in static conditions. In addition, a trend where higher lipid concentrations induced longer-lasting bilayers has also been reported, with the self-assembly process (i.e. from multilamellar to bilayer) of lipids at the droplet interface taking longer to occur for higher lipid concentration^18^. In our system instead, if coalescence did not occur within seconds after contact between droplets, DIB coalescence was never observed.

In static or ‘slow’ dynamic conditions, droplets coalescence can be avoided by sufficiently increasing the phospholipid concentration thus ensuring phospholipid coverage at a droplet interface^1^. Differently, for ‘fast’ dynamic conditions, the lipid coverage at a droplet interface depends also on the hydrodynamic forces and the aqueous solutions used^29^,^30^. On one side, the faster a droplet moves, the greater the lipid adsorption rate at its interface^31^. On the other, lipids at the front of a travelling droplet tend to be displaced to the back of the droplet interface, creating a gradient of interfacial tension and leading to lipid depletion at the front of the droplet^32^. This localised decrease in lipid concentration can induce coalescence upon collision of phospholipid stabilised W/O droplets. The dynamics of emulsion stability has been previously investigated in rectangular microchannels^33^,^34^. Both studies have described the relationship between the rate of droplet coalescence and surfactant concentration depending on the size and dispersion of a group of colliding droplets within a microfluidic chamber. Additionally, previous reports^29^,^35^ have also described coalescence effects in microdroplets when fast fluid dynamics induce a local depletion of the surfactant at the interface between two contacting droplets. Finally, droplet stabilization in ‘very fast’ flow rates (i.e. twice the magnitude than in our system) has also been investigated^31^. For the microchannel geometry used and a continuous train of adjacent droplets, a trend was identified where higher droplet velocities reduced the coalescence rate of phospholipid stabilised droplets. Overall, regardless of the microfluidic geometry, it can be concluded that to avoid droplet coalescence it is necessary to ensure that the lipid adsorption dynamics at a W/O interface are faster than the lipid depletion dynamics caused by interfacial shear before droplet collision.

**Figure 5:**
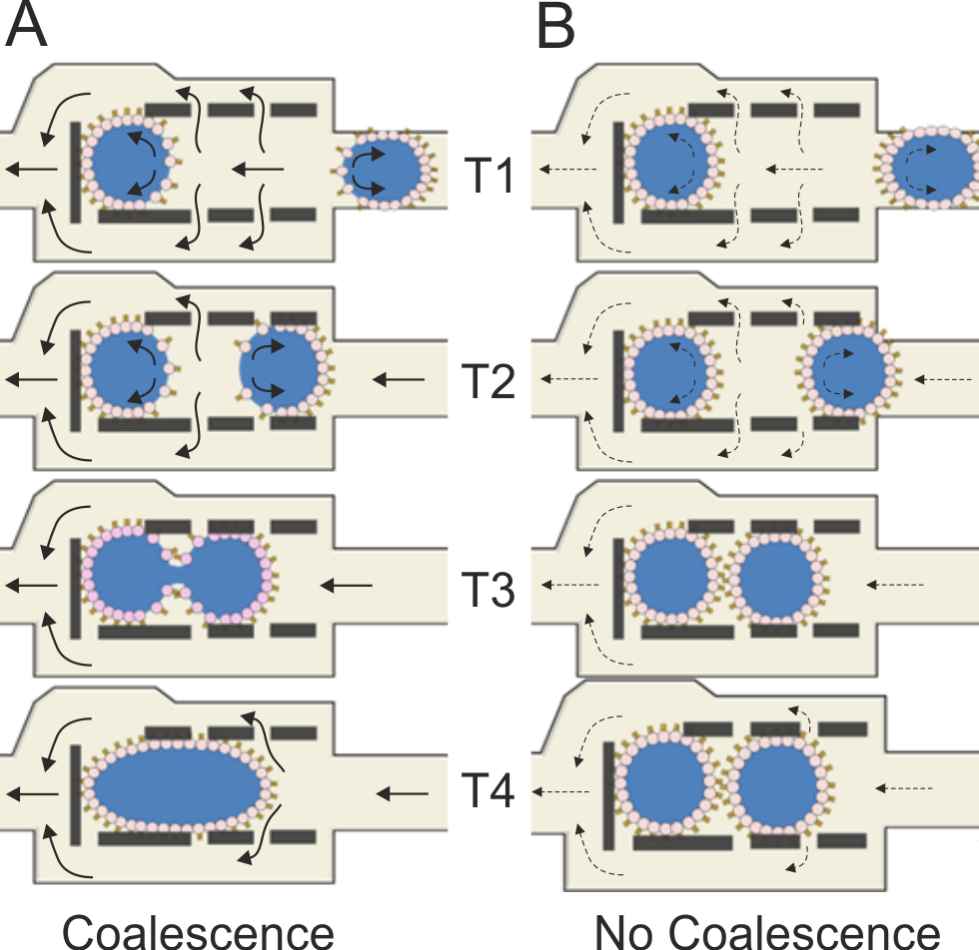
Schematic representation of coalescence (A) and non-coalescence (B) mechanisms of psW/O droplets within a shift register due to a difference in droplet velocity (solid arrows, velocities above threshold; dashed arrows, velocities below threshold with respect to graph in Figure 2). During normal device operation, when a droplet reaches an empty shift register it remains trapped within the pillar structure if the correct balance between the hydrodynamic pressure difference between the inlet and outlet points of the register and the Laplace pressure of the droplet interface is achieved. Because the oil is flowing within the register and exiting through the gaps between the pillars, the interface of a trapped droplet can be depleted of phospholipids proportionally to the magnitude of the shear forces parallel to the oil flow directions. When a second droplet, presenting a phospholipid-depleted front interface, enters the register, the oil layer between the two drops is drained through the pillars and contact between the two psW/O droplets will occur. Depending on the phospholipid adsorption dynamics, (i.e. phospholipid concentration and magnitude of hydrodynamic forces), the two droplets will coalesce depending on the degree of phospholipid coverage at both droplet interfaces.

Specific to this work, Figure 5 shows schematically the two possible scenarios that can occur during a droplet arraying process within a shift register. In both cases, we consider the first droplet as static (already trapped in a register), while the second is approaching and eventually contacts the first to form a DIB. Both droplets are exposed to interfacial shear stress caused by the drainage of the interstitial oil (through the gap between pillars in a shift register structure - arrows in Figure 5), which occurs as the moving droplet approaches the resting one. If droplet velocity is used as a measure of the shear stress at a droplet interface moving in a rectangular channel, a higher velocity corresponds to greater shear^30^, causing a more pronounced change in the phospholipid distribution at the droplet interface. Consistent with what was discussed above, a high shear stress induces a rapid depletion of the phospholipid film, rendering emulsion interfaces prone to coalescence (Figure 5A). Successful formation of DIB occurs when lipid adsorption dynamics are dominant over interfacial phospholipid depletion, thus preventing coalescence. When analysing the magnitudes of the velocities of psW/O droplet trains in rectangular microchannels in the literature^15^,^16^, similar velocity values to those identified in Figure 2 are obtained, suggesting that the considerations outlined above are generally applicable to phospholipid stabilised emulsions at the microscale. In conclusion, the correlation between droplet velocity, lipid concentration and coalescence can be identified for a specific lipid mixture and this information then used for instructing the design of the channel network.

### Fluorescent assays

The importance of carrying out permeation assays *in vitro* and both the qualitative and quantitative estimation of ion-channel mediated molecular transport is well documented^25^,^36^,^37^. A system that can allow such assays to be performed in an automated and customisable manner can therefore find applications in the pharmacokinetic screening of compounds during the drug development process. Here, proof-of-concept data is shown estimating the permeability of fluorescein through microfluidic DIBs and ion transport across α-haemolysin bearing DIBs. To evaluate the permeation of substances from a donor droplet to an acceptor droplet, we used the apparent permeability index *P_app_*^36^, which was calculated using the adapted equation (1): 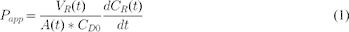 where *C_D0_* is the initial concentration of the compound in the donor droplet, *C_R_(t)* is the concentration of the compound in the acceptor droplet over time, *A(t)* is the estimated total surface area of the DIB (i.e. taking into account that some droplets had two DIBs) and *V_R_(t)* is the estimated volume of the acceptor droplet over time. The droplet volumes and DIB surface areas at each time point were estimated from the respective microscopy images (approximating droplets to ellipsoids and DIB areas to ellipses). The calculation takes into account variability in droplet size and bilayer area for all measurements, therefore temporal changes do not affect the estimate of the permeability coefficient. As the fluorescence intensity is proportional to molecular concentration, the intensity values were used as a proxy for compound concentration. The estimated *P_app_* value for fluorescein at pH 7.4 was 2.01×10^−6^ ± 1.46×10^−6^ cm/s (calculated from 5 different experiments) which falls within the range reported in the literature between 1.6×10^−6^ cm/s and 21.2×10^−6^ cm/s^25^,^37^,^38^ according to the pH used.

The ability of fluorescein, but not calcein, to traverse the DIB confirmed the formation of a selectively permeable lipid interface. However, it is important to note that dye leakage experiments do not prove the existence of a lipid bilayer (whilst the flow of ions through α-haemolysin pores does). Previous reports have in fact shown that the interactions between lipid molecules and dyes are dependent on the number of lipid layers (or lipid multilayer), with leakage time being directly proportional to the number of lipid layers^39^. The estimated *P_app_* value for Ca^2+^ ion across α-haemolysin pores was 7.08×10^−6^ ± 1.76×10^−6^ cm/s (calculated from 3 different experiments) for the concentration of monomers used. This approach can provide an insight into the transport dynamics through ion channels when, for instance, determining the effects on ion flux through proteins caused by blocking agents^17^. To quantify the concentration of calcium ions within an acceptor droplet at different time points, a calibration curve was also produced (Figure S7 in ESI).

The ability to qualitatively (and potentially quantitatively) identify DIB permeation values demonstrates the suitability of our system for investigating processes occurring across an artificial lipid bilayer in a miniaturised and scalable format.

## Conclusions

We have developed and validated a microfluidic network based on pressure driven flow, capable of stable and robust psW/O droplet generation, alternation and trapping within register elements for the formation of a series of DIB arrays of desired length and their storage. First, we have identified an important relationship between the droplet velocity and lipid concentration which identifies the conditions to prevent droplet coalescence without hindering the microfluidic functionalities of our system. Then, we have discussed how the scalability and throughput of results depends on the serial connectivity of the register network and the dynamics of mass transport occurring across a DIB. Finally, we have demonstrated the successful formation of lipid bilayers, providing proof-of-concept results of passive molecular permeation and ion-channel mediated permeation of molecules and ions through DIBs using fluorescent assays. The proposed system can lead to new platforms for higher-throughput and miniaturised version of parallel artificial membrane permeability assays (PAMPA) and being applied to large-scale studies of processes occurring across artificial lipid bilayers, such as molecular transport^8^, fusogenic properties of peptides^40^ and virus transport and fusion^41^.

Furthermore, the architecture has also potential to be utilised for drug discovery assays using non self-inserting eukaryotic transmembrane proteins. However, these are typically non water-soluble proteins and require a micellar or liposome environment to maintain functionality before reconstitution into a DIB. This is a challenging task and further improvement, both procedural and technological, are required, for instance, by optimising protocols for either cell free expression of transmembrane proteins^42^ or proteoliposome delivery^14^ in droplet-based systems. The integration of microelectrodes is also possible which will allow for large scale electrophysiology readouts^43^,^44^, allowing more sensitive assays to be performed than those obtained with fluorescent dye reporters. However, this approach increases the complexity associated with system microfabrication and costs. Finally, the integration of on-demand droplet production^45^ with multiple inlets may enable the automated and combinatorial arraying of DIB networks to be obtained for testing multiple compounds within one device.

## Author Contributions

The proposed work was carried out by BS under supervision of MZ and equal contribution has been made in planning experiments, writing and reviewing this manuscript.

## Supplementary Material

Supplementary Information Supplementary information doc

Supplementary Information SI video 1A

Supplementary Information SI video 1B

Supplementary Information SI video 1C

Supplementary Information SI video 2

Supplementary Information SI video 3

Supplementary Information SI video 4

Supplementary Information SI video 5
