# Supplementary material for: Droplet-interface-bilayer assays in microfluidic passive networks
Source: Sci Rep. 2015 Apr 24;5:9951. doi: 10.1038/srep09951 (PMC4408985; doi:10.1038/srep09951)
Supplement: Supplementary Information — doc [file srep09951-s1.pdf]

Electronic Supplementary Information (ESI)

Droplet-interface-bilayer assays in microfluidic passive networks

Bárbara Schlicht and Michele Zagnoni*

Centre for Microsystems and Photonics, Electronic and Electrical Engineering, University of Strathclyde, Glasgow, G1 1XW, UK

**email address: michele.zagnoni@strath.ac.uk*

MOVIES

Movie S1A, S1B & S1C: Movies showing device operation. Phases are 10 mM HEPES, 200 mM KCl for the aqueous phase and 5mg/ml of DPhPC in hexadecane for the oil phase. Movie S1A shows droplet formation and droplet alternation at a Y-Junction. Movie S1B shows the channel structure and a by-pass channel were the oil phase is diverted to reduce droplet velocity before the register area thus preventing coalescence. In this instance, the droplet velocity has been halved after the bypass channel. Movie S1C shows droplet trapping and arraying without coalescence within droplet shift registers. Movie has been compressed with Xvid codec. All three videos are played at twice the real time speed.

Movie S2, S3 & S4: Movies show how droplet coalescence is dependent on their velocity for the same lipid concentration when droplets contact each other within shift registers due to changes in phospholipid distribution at the W/O interface of contact. The aqueous phase was 10 mM HEPES, 200 mM KCl, 5mg/ml asolectin, the oil phase was hexadecane. Movie S2 (droplet travelling at 978 µm/s) and S3 (droplets traveling at 255 µm/s) show droplet coalescence. Movie S4 (droplets traveling at 115 µm/s) show no droplet coalescence. All three videos are played in real time. Movies have been compressed with Xvid codec.

Movie S5: Time lapse of fluorescence increase in acceptor droplets due to Ca^{2+} flux from donor droplets, due to facilitated diffusion of calcium ions through α-haemolysin ion channels incorporated into DIBs. Acceptor droplets contained 250 µM Fluo-8, 10mM HEPES, 333 µM EDTA, 2M KCl and donor droplets contained 2 µg/ml α-Haemolysin, 10mM HEPES, 20 µM EDTA, 1M $CaCl_2$. Movie has been compressed with Xvid codec.

FIGURES

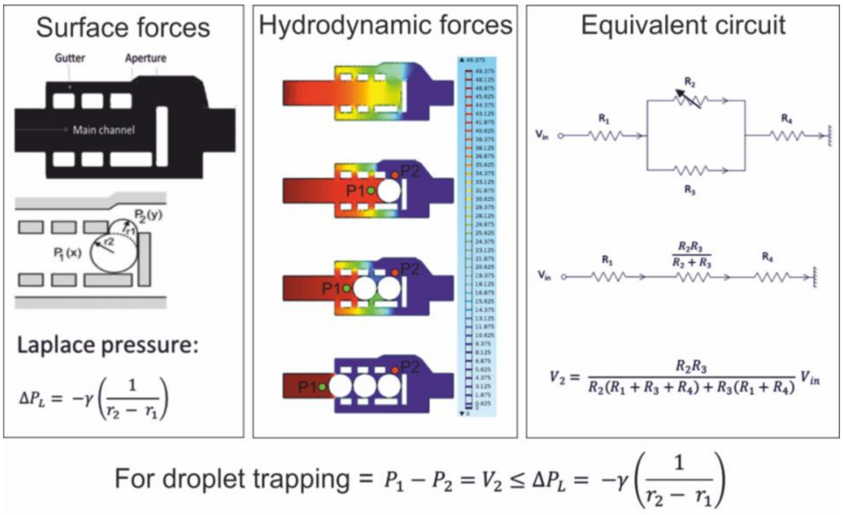

Figure S1. Working principle of a microfluidic droplet shift register[1]. (left) The shift register element consists of three channels (a central channel and two side channels or gutters) separated by pillars. The layout is designed to induce droplets to flow through the central channel, while the oil phase also flows in the two side gutters. After a register element is filled with the continuous phase, a droplet arriving at the element is trapped in the central branch if the differential hydrodynamic pressure between the upstream (P_1, at the point x) and the downstream side (P_2, at the point y) of the drop is lower than the Laplace pressure generated at the outlet of the register. (centre) Numerical simulations of the Navier-Stokes equations for a shift register geometry with an increasing number of droplets trapped within the register. Any additional droplet arriving after the first at the upstream side of the element arrayed along its length. Colour shows a steady increase in differential pressure (P_1-P_2) for a higher number of droplets trapped. When the register length is filled with droplets, an additional one forces the drop at the downstream side to exit the register, creating a serial shift of the drops stored within the pillars. (right) Simplified model of a register element using an equivalent electric circuit. Each droplet present in the register changes the value of the fluidic resistance of the central channel within the pillar area, causing the overall resistance of the channel to increase, where V_{in} represents the constant pressure applied to drive the fluidic phases, R_1 is the resistance from the inlet to the start of the register, R_2 is the resistance in the main channel which varies depending on how many droplets have been trapped, R_3 represents the resistance from the two gutters, R_4 denotes the resistance from the end of the register to the outlet and V_2 is the differential pressure across the trapped droplets. Therefore, a trend develops between interfacial tension (directly related to lipid concentration) and differential pressure across a register for trapping efficiency, as described by the equation above.

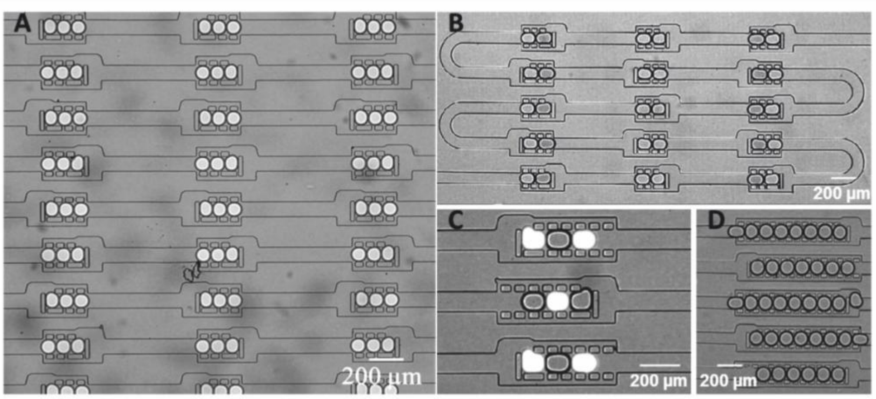

Figure S2. Examples of a variety of droplet shift register structures to create different sizes of DIB arrays. (A) Image show large scale DIB production with a droplet shift register array. (B, C & D) Bright field and

superimposed fluorescent images show formation of different lengths of DIB arrays. In all the figures, shift register structures are connected in series.

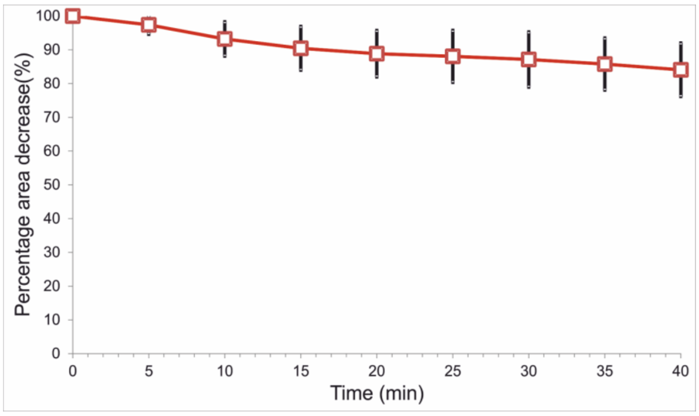

Figure S3. Droplet shrinkage within registers. The graph shows droplet shrinkage as a function of time. The error bars represent the standard deviation resulting from a set of 15 measurements. After 40 minutes, a 16% reduction in droplet area was observed when using devices pre-soaked in aqueous media before use.

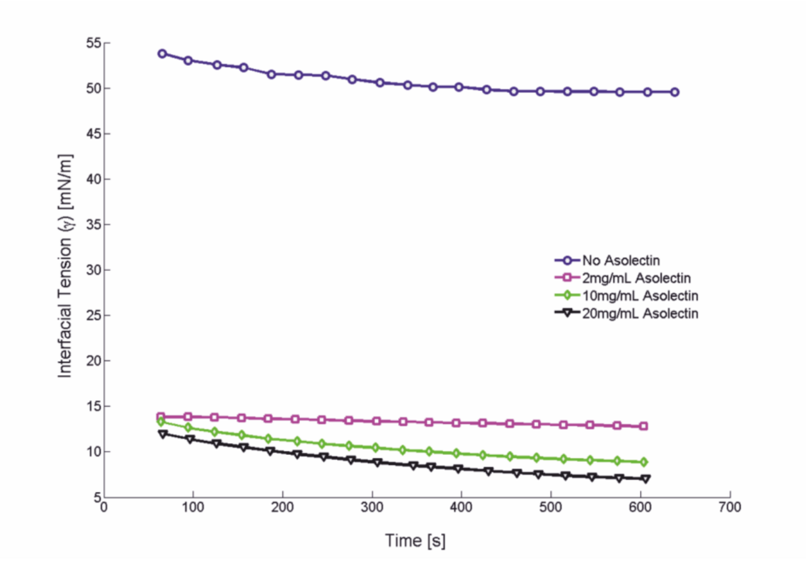

Figure S4. Temporal measurements of interfacial tension using the pendant drop method for different concentrations of asolectin in hexadecane. Buffer was 10 mM HEPES, 200 mM KCL and the oil phase was asolectin in hexadecane. The decreasing trend is due to adsorption of lipid molecules (present in micellar form in the hexadecane) to the drop interface by diffusion. The higher the lipid concentration in hexadecane, the lower the interfacial tension.

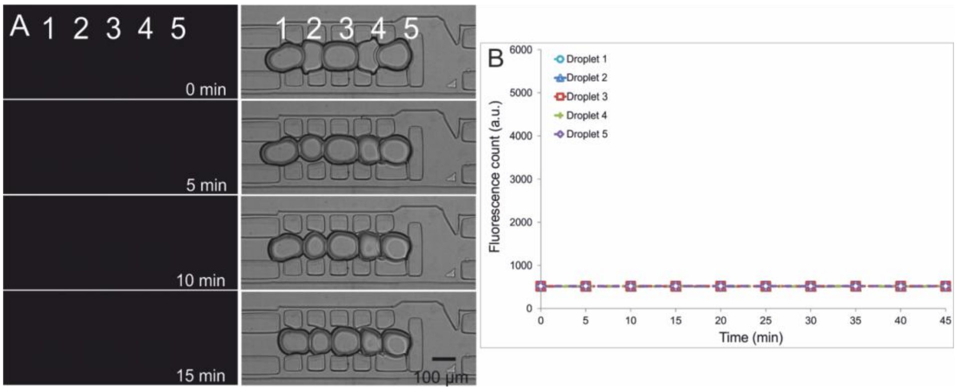

Figure S5. Control experiment for Figure 4 in the manuscript. Droplets in ABABA configuration contained 10mM HEPES, 20 µM EDTA, 1M CaCl2, at pH 7.4 (donor droplet) and 10mM HEPES, 333 µM EDTA, 2M KCl, 250 µM Fluo-8, at pH 7.4 (acceptor droplet). (A) The left hand column shows fluorescence microscopy images taken using a fluorescein isothiocyanate (FITC) filter; the corresponding bright field can be seen in the right hand column. (B) When no α-Haemolysin monomers were encapsulated in the droplets, no diffusion of calcium ions occurred across the DIBs. Experiments were carried out for 45 minutes with no detectable increase in fluorescent signal.

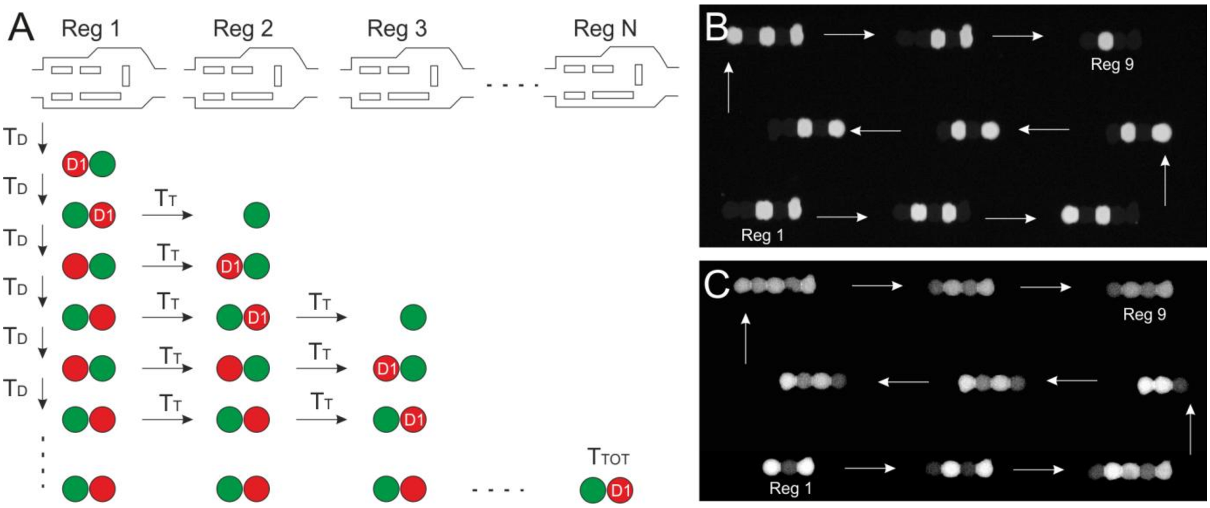

Figure S6. Trade-off between the dynamics of mass transport across a DIB and the device operation. (A) Schematic shows *N* droplet shift registers in series, each of which can store a maximum of two droplets. As an example, the temporal sequence of a droplet *D1* starting from the first register and reaching the Nth register is analysed. In steady state condition of operation, a droplet will reach a new shift register at every T_D interval of time and will take T_T time to travel from one register to the next, resulting in a total time of $\boldsymbol{T_{TOT} = (2 * N * T_D + (N - 1) * T_T)}$ to completely fill the *N* registers (i.e. from N empty registers to N full registers). By the time all *N* registers are full, due to the serial connection between the registers, *D1* droplet would have been in touch with any adjacent droplet for a time $\boldsymbol{T_C = N * T_D}$ during the filling procedure. (B) Fluorescence microscopy image showing nine full registers with alternating droplets containing buffer and calcein. As the time associated with the transport dynamics of calcein ($T_{P\text{-calcein}}$) across a DIB are much longer than T_{TOT}, all nine registers can be monitored in parallel. (C) Fluorescence microscopy image showing the same 9 full registers with alternating droplets containing buffer and fluorescein. As the time associated with the transport dynamics of fluorescein ($T_{P\text{-fluorescein}}$) across a DIB are shorter than T_{TOT}, mass transfer across DIBs are negligible for a time necessary to fill approximately the first two registers only, whereas from the third register onwards it is apparent that considerable exchange of fluorescein had already occurred.

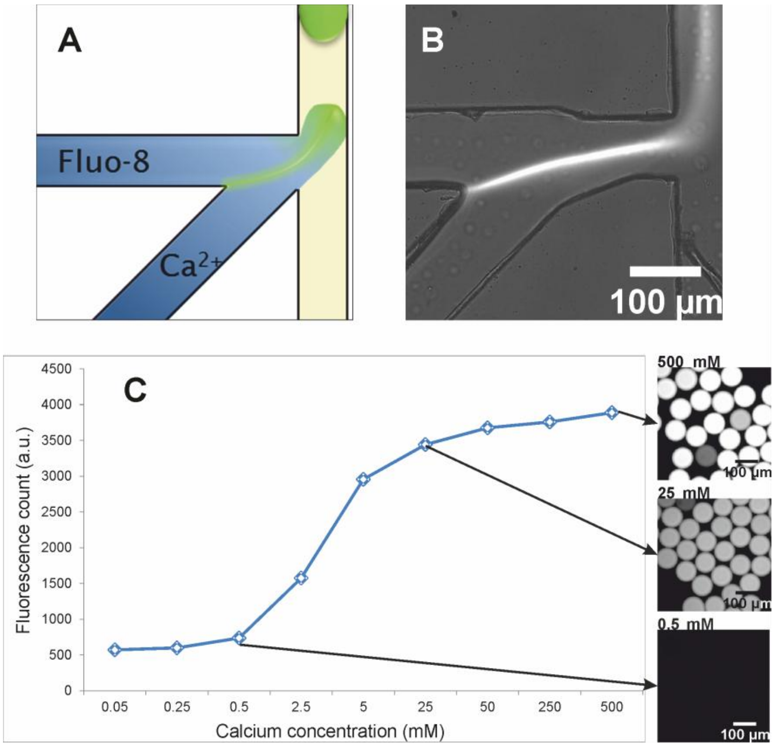

Figure S7. Calibration curve for quantitative analysis of calcium transport across DIBs. Schematic representation (A) and fluorescent microscope image (B) of a T-junction where two streams containing Fluo-8 dye solution (250 µM Fluo-8, 10 mM HEPES, 333 µM EDTA, 2M KCl at pH7.4) and calcium buffer (10 mM HEPES, 20 µM EDTA, 100 µM - 1M $CaCl_2$ at pH 7.4) were mixed and emulsified. Due to laminar flow property, only a thin fluorescent interface is instantaneously formed before droplet production. Fluo-8 and Ca^{2+} solutions (in a range 100 µM to 1M $CaCl_2$) where mixed in a 50:50 ratio to produce fluorescent droplets which were subsequently stored in a large compartment downstream. (C) The fluorescence intensity of 10 droplets was measured for each concentration tested and the average value was used to produce a calibration curve which can be used to estimate the concentration of calcium within droplets during α-Haemolysin experiments. The assay detection sensitivity was estimated as 250 µM Ca^{2+} concentration in droplets for the phases used.

References

1. Zagnoni, M.; Cooper, J. M., A microdroplet-based shift register. *Lab Chip* **2010,** *10* (22), 3069-3073.
